# Supplementary material for: Enhanced Behavioral Performance through Interareal Gamma and Beta Synchronization
Source: bioRxiv. 2023 Mar 6:2023.03.06.531093. Preprint. [Version 1] doi: 10.1101/2023.03.06.531093 (PMC10028832; doi:10.1101/2023.03.06.531093)
Supplement: Supplement 1 [file NIHPP2023.03.06.531093v1-supplement-1.pdf]

bioRxiv preprint doi: https://doi.org/10.1101/2023.03.06.531093; this version posted March 6, 2023. The copyright holder for this preprint (which was not certified by peer review) is the author/funder, who has granted bioRxiv a license to display the preprint in perpetuity. It is made available under aCC-BY 4.0 International license.

Figure S1. Site-pair selection based on phase coherence. Related to Figures 2 and 3.

(A, B) PPC for all interareal site pairs between V1 and V4 (A) or F1 and F4 (B) of monkey K, averaged over conditions IN and OUT. Site pairs with a PPC crossing a threshold (mean+3SD of all PPC values across all frequencies for all site pairs of all recorded areas; dashed lines) were selected for further analyses and are labeled with stars. Insets show zoom-ins for individual selected site pairs from V1-V4 (A) and F1-F4 (B), respectively.

Figure S2. Interareal synchronization in an occipital and fronto-central cluster, in gamma and beta frequencies. Related to Figures 2 and 3.

(A, B) PPC averaged over selected site pairs of both monkeys in each area pair, aligned to the individual gamma (A) or beta (B) peak frequency. Areas were ordered according to hierarchical level. We selected area pairs with PPC values exceeding a threshold (mean+2SD of all averaged PPC values across all frequencies and all area pairs). Dashed squares show the occipital gamma cluster (A) and the fronto-central beta cluster (B). The area pair DP-7A showed high beta PPC, but was not a direct neighbor of the beta cluster.

Figure S3. Across-trial correlation between power spectra and RTs. Related to Figure 3.

(A, B) Across-trial correlation between power spectra and RTs for IN (red) and OUT (black) conditions, aligned to the gamma peak frequency in the occipital cluster (A) and the beta peak frequency in the fronto-central cluster (B). Correlations were first calculated per site and then averaged over sites.

Figure S4. Occipital gamma GC correlates with fronto-central beta GC during IN condition. Related to Figure 5.

(A, B) Jackknife correlation (see Methods for details) between occipital gamma GC and fronto-central beta GCs, separately for condition IN (A) and OUT (B). Each dot represents the respective GC values from one jackknife replication, that is, after leaving out one trial, averaged over site pairs of the corresponding areas (gamma GC in occipital areas and beta GC in fronto-central areas).

(C) Comparison of the empirically observed difference in GC correlation (IN-OUT) with chance distribution based on 1000 randomizations of trials.

Figure S5. Similar effects for frontal cluster as for fronto-central cluster.

(A) Average PPC between area pairs in the frontal cluster (F1, F2, F3; inset), for IN (red) versus OUT (black) conditions. Shaded areas indicate SEM across site pairs. Black horizontal line indicates frequencies with a significant difference between IN and OUT conditions.

(B) Interareal GC, averaged over all site pairs of the frontal cluster (inset), aligned to the gamma peak, separately in the bottom-up (BU, tick lines) and top-down (TD, narrow lines) directions, and for the IN (red) and OUT (black) condition. Colored horizontal lines denote significant differences between conditions (IN, OUT, BU, TD), as indicated in the color legend.

bioRxiv preprint doi: https://doi.org/10.1101/2023.03.06.531093; this version posted March 6, 2023. The copyright holder for this preprint (which was not certified by peer review) is the author/funder, who has granted bioRxiv a license to display the preprint in perpetuity. It is made available under aCC-BY 4.0 International license.

(C) GC between frontal and occipital clusters aligned to beta peak GC, for IN (red) and OUT (black) conditions, and in bottom-up (BU, tick lines) and top-down (TD, narrow lines) directions, separately. Colored horizontal lines indicate significant frequency bands for the indicated comparisons.

(D) Correlation between GPRs and RTs, as a function of frequency for IN (red) and OUT (black) conditions. Black horizontal dashed lines indicate significance thresholds, corrected for multiple comparisons.

(E) Jackknife correlation (see Methods for details) between single-trial beta GCs and RTs between the frontal cluster for IN (red) and OUT (black) conditions and in the bottom-up (BU, tick lines) and top-down (TD, narrow lines) directions.

(F) Jackknife correlation between single-trial beta GCs and RTs between the occipital and frontal cluster for IN (red) and OUT (black) conditions and in the bottom-up (BU, tick lines) and top-down (TD, narrow lines) directions.

Figure S6. Information flow between fronto-central areas. Related to Figure 7.

(A) GC between the area pairs in the fronto-central cluster for bottom-up (BU, tick line) and top-down (TD, narrow line) directions, aligned to the beta peak. Areas were ordered according to hierarchical level. The area pairs highlighted in gray are between areas of different brain systems, namely the somatosensory (S1, 5) and frontal (F1, F2, F4) system.

(B) Strength of beta GC between somatosensory and between frontal areas. Between the recorded somatosensory areas, S1 and area 5, beta-band GC was stronger in the top-down direction. Between the recorded frontal areas, F1, F2, F4, beta-band GC was stronger in the bottom-up direction. The strength of GC is indicated by the thickness of the connecting lines.

bioRxiv preprint doi: https://doi.org/10.1101/2023.03.06.531093; this version posted March 6, 2023. The copyright holder for this preprint (which was not certified by peer review) is the author/funder, who has granted bioRxiv a license to display the preprint in perpetuity. It is made available under aCC-BY 4.0 International license.

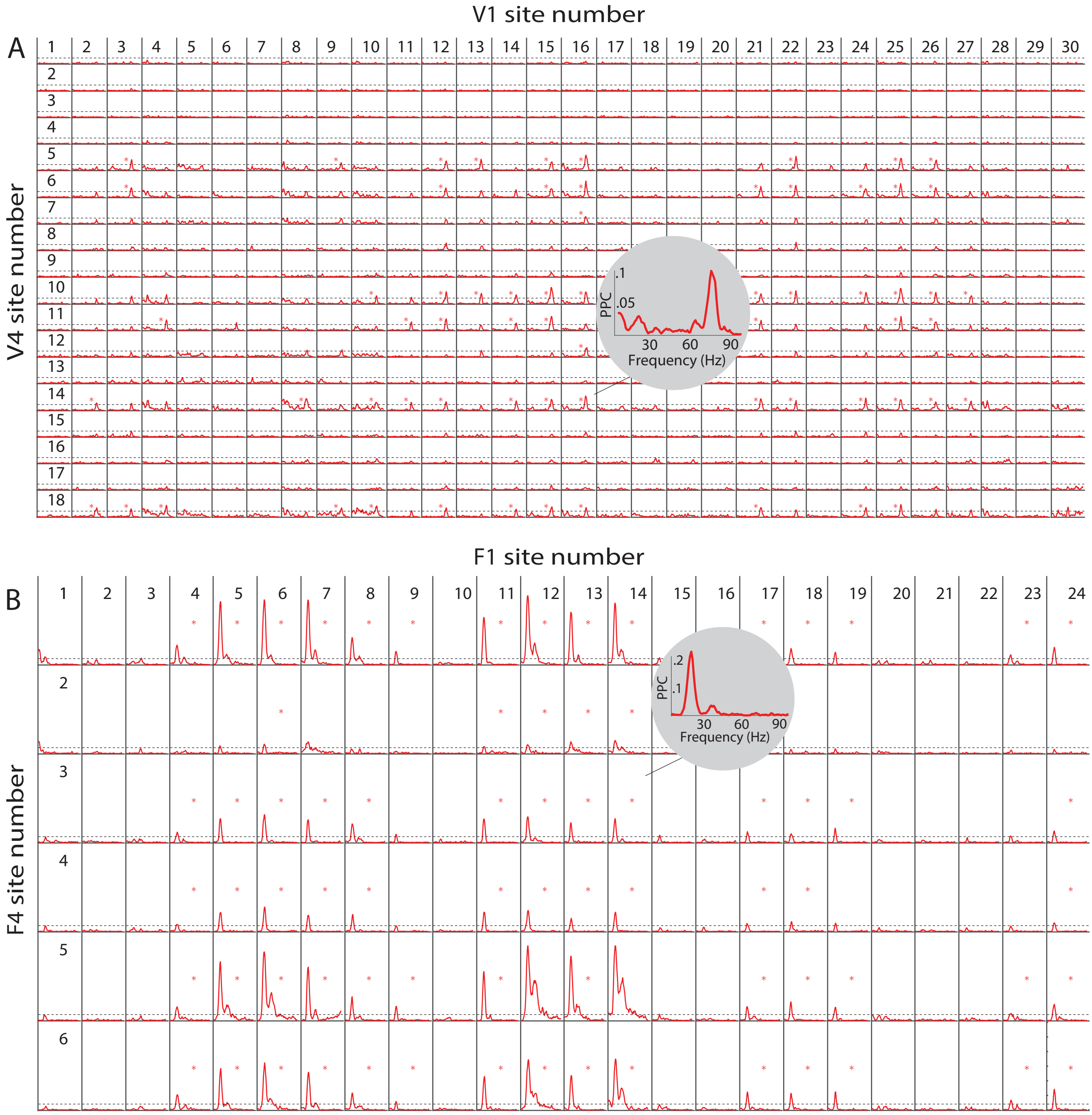

Supplementary figure 1

bioRxiv preprint doi: https://doi.org/10.1101/2023.03.06.531093; this version posted March 6, 2023. The copyright holder for this preprint (which was not certified by peer review) is the author/funder, who has granted bioRxiv a license to display the preprint in perpetuity. It is made available under aCC-BY 4.0 International license.

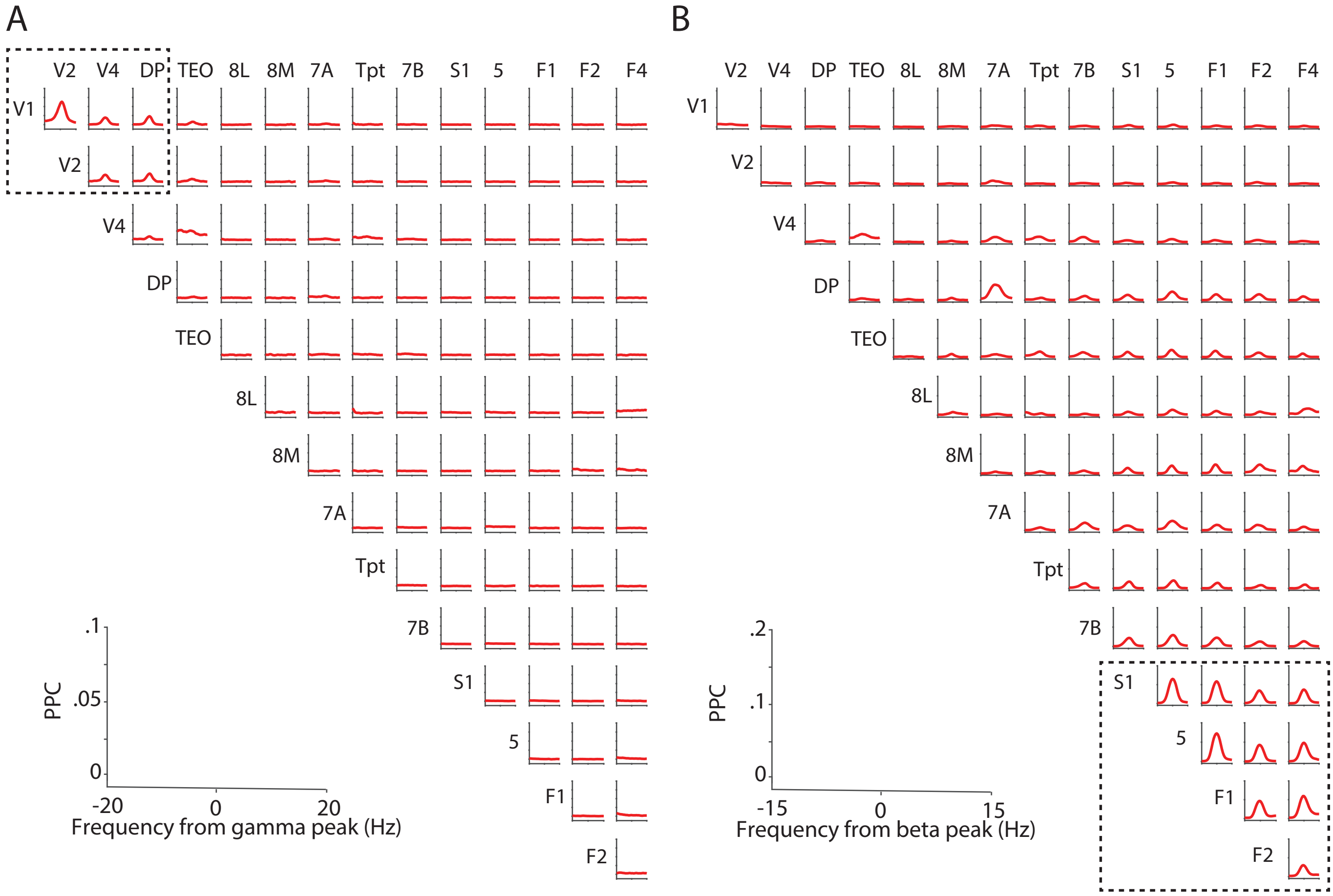

Supplementary figure 2

bioRxiv preprint doi: https://doi.org/10.1101/2023.03.06.531093; this version posted March 6, 2023. The copyright holder for this preprint (which was not certified by peer review) is the author/funder, who has granted bioRxiv a license to display the preprint in perpetuity. It is made available under aCC-BY 4.0 International license.

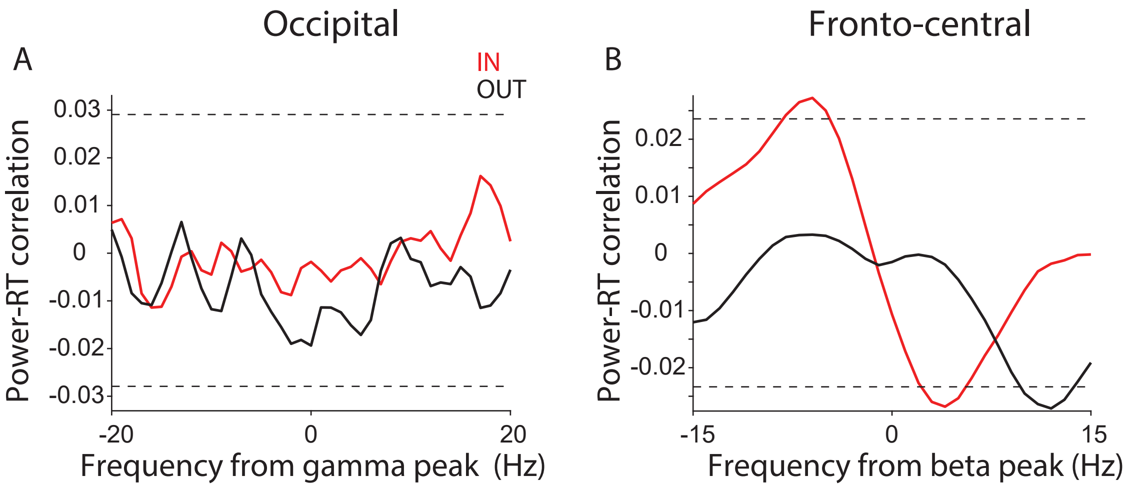

Supplementary figure 3

bioRxiv preprint doi: https://doi.org/10.1101/2023.03.06.531093; this version posted March 6, 2023. The copyright holder for this preprint (which was not certified by peer review) is the author/funder, who has granted bioRxiv a license to display the preprint in perpetuity. It is made available under aCC-BY 4.0 International license.

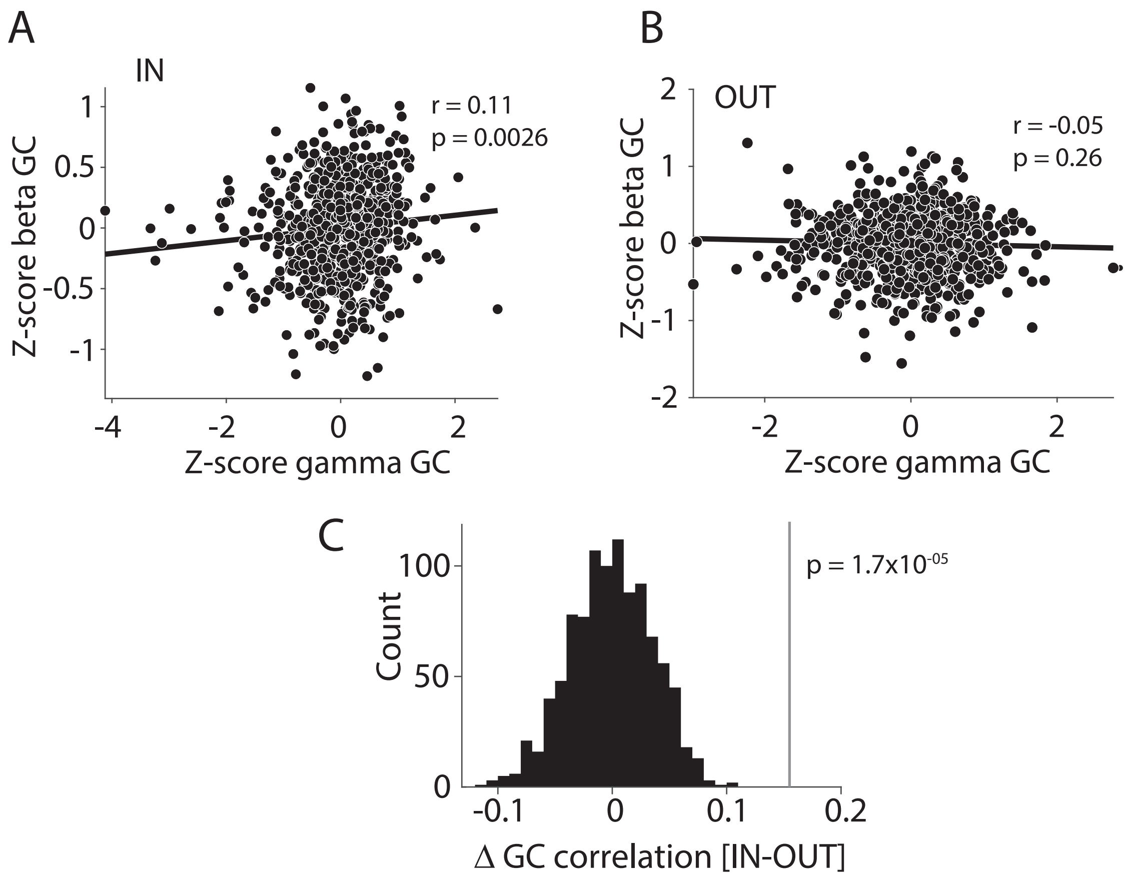

Supplementary figure 4

bioRxiv preprint doi: https://doi.org/10.1101/2023.03.06.531093; this version posted March 6, 2023. The copyright holder for this preprint (which was not certified by peer review) is the author/funder, who has granted bioRxiv a license to display the preprint in perpetuity. It is made available under aCC-BY 4.0 International license.

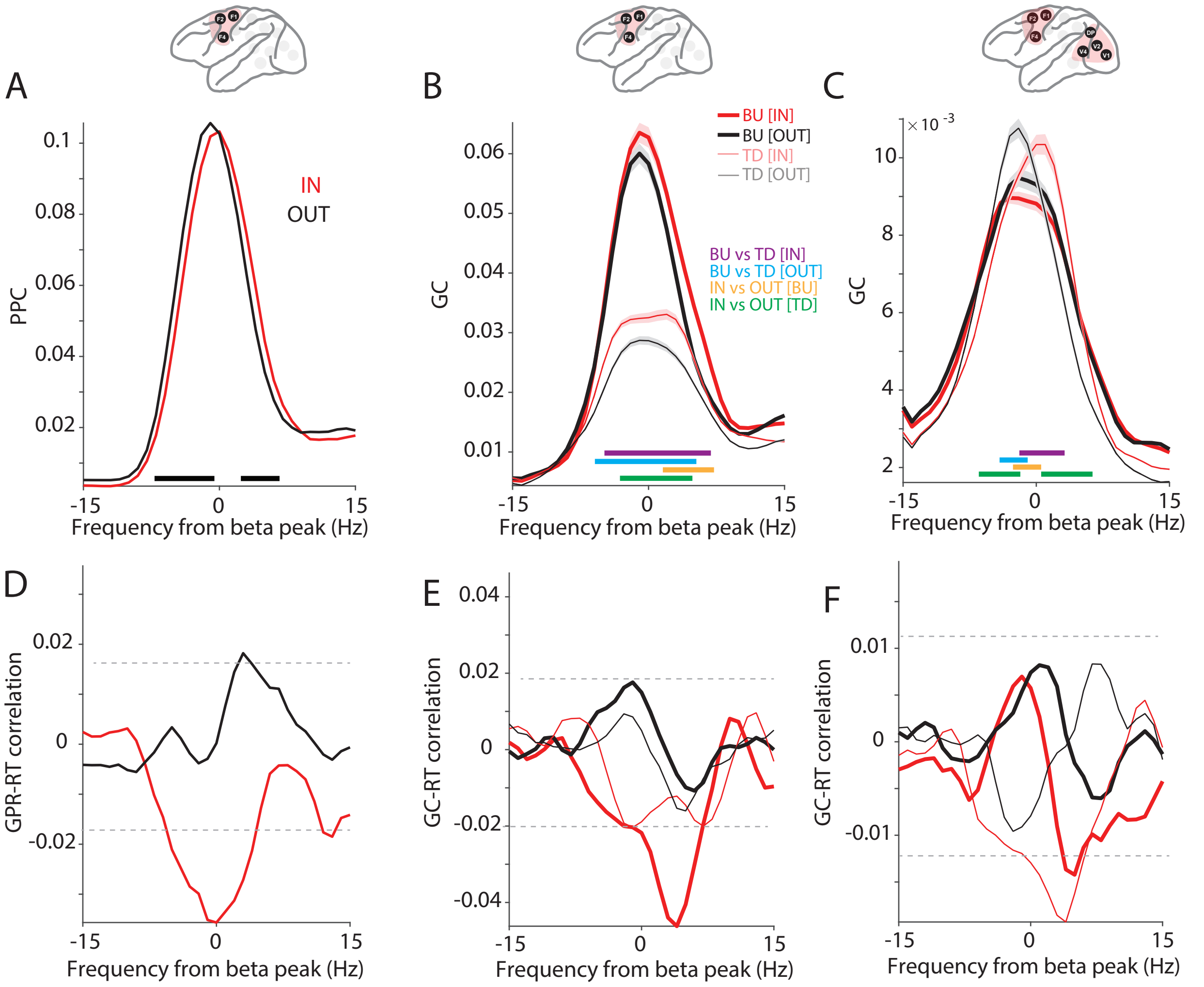

Supplementary figure 5

bioRxiv preprint doi: https://doi.org/10.1101/2023.03.06.531093; this version posted March 6, 2023. The copyright holder for this preprint (which was not certified by peer review) is the author/funder, who has granted bioRxiv a license to display the preprint in perpetuity. It is made available under aCC-BY 4.0 International license.

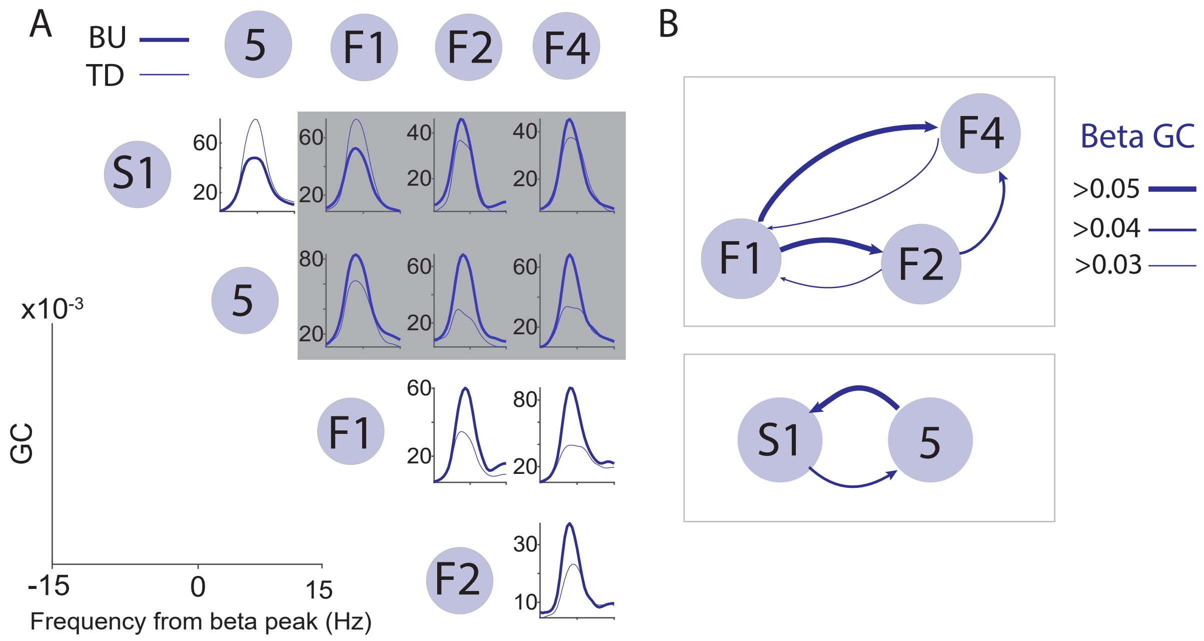

Supplementary figure 6
